# A public health threat in Hungary: obesity, 2013

**DOI:** 10.1186/1471-2458-14-798

**Published:** 2014-08-05

**Authors:** Imre Rurik, Péter Torzsa, Judit Szidor, Csaba Móczár, Gabriella Iski, Éva Albók, Tímea Ungvári, Zoltán Jancsó, János Sándor

**Affiliations:** Department of Family and Occupational Medicine, Faculty of Public Health, University of Debrecen, Móricz Zs. krt.22, 4032 Debrecen, Hungary; Division of Biostatistics and Epidemiology, Department of Preventive Medicine, Faculty of Public Health, University of Debrecen, Debrecen, Hungary; Department of Family Medicine, Faculty of Medicine, Semmelweis University, Budapest, Hungary; Irinyi Primary Health Care Center, Kecskemét, Hungary

**Keywords:** Hungary, Obesity, Overweight, Prevalence, Primary care, Public health

## Abstract

**Background:**

In Hungary, the last wide-range evaluation about nutritional status of the population was completed in 1988. Since then, only limited data were available. Our *aim* was to collect, analyze and present updated prevalence data.

**Methods:**

Anthropometric, educational and morbidity data of persons above 18 y were registered in all geographical regions of Hungary, at primary care encounters and within community settings.

**Results:**

Data (BMI, waist circumference, educational level) of 40,331 individuals (16,544 men, 23,787 women) were analyzed. Overall prevalence for overweight was 40.4% among men, 31.3% among women, while for obesity 32.0% and 31.5%, respectively. Abdominal obesity was 37.1% in males, 60.9% in females. Among men, the prevalence of overweight-*obesity* was: under 35 y = 32.5%-*16.2%,* between 35-60 y = 40.6%-*34.7%,* over 60 y = 44.3%-*36.7%.* Among women, in the same age categories were: 17.8%-*13.8%,* 29.7%-*29.0%, and* 36.9%-*39.0%.* Data were presented according to age by decades as well. The highest odds ratio of *overweight (OR: 1.079; 95% CI [1.026-1.135])* was registered by middle educational level, the lowest odds ratio of *obesity (OR: 0.500; 95% CI [0.463-0.539])* by the highest educational level. The highest proportion of obese people lived in villages (35.4%) and in Budapest (28.9%). Distribution of overweighed persons were: Budapest (37.1%), other cities (35.8%), villages (33.8%). Registered metabolic morbidities were strongly correlated with BMIs and both were inversely related to the level of urbanization. Over the previous decades, there has been a shift in the distribution of population toward being overweight and moreover obese, it was most prominent among males, mainly in younger generation.

**Conclusions:**

Evaluation covered 0.53% of the total population over 18 y and could be very close to the proper national representativeness. The threat of obesity and related morbidities require higher public awareness and interventions.

## Background

Obesity, a worldwide pandemic, is well-known for the readers who are interested in metabolic diseases with high public health impact. While obesity is mainly a medical problem, its related metabolic, cardiovascular and other diseases have serious public health and other economic or social implications. Health care services provided for obese patients are usually more expensive, mainly for the complications related to this condition. The increasing ratio of overweight and obese people is visible in all health care settings and also in public places. It has been described as a world-wide trend, although there are differences between and within countries and populations [1, 2].

A similar trend was observed in previous Hungarian evaluations, although the issue and importance of obesity were realized later than in other countries [3, 4].

Measuring and registering anthropometric parameters is rarely obligatory at different levels of health care provision, let alone in the “healthy” population. Although measuring everyone in a society is impossible, finding and reaching the required representation is crucial. Studies and surveys in other countries have used quite different methods for the selection of participants and so have the Hungarian ones [1, 5]. Representative selections can be based on geographical or domicile distribution; they may respect the age cohorts and educational levels. Planning and performing a large scale and, also, representative evaluation often involves compromises.

The first wide range Hungarian study was performed between 1985 and 1988. It was a professionally planned survey with wide geographical distribution, targeting the whole population of the country. Morbidities, anthropometric parameters, nutritional habits and living circumstances were registered. Data of *16,641* individuals (*7,042* men and *9,599* women) were collected by trained staff of the National Institute of Food Hygiene and Nutrition [3]. The following means of BMI were found within the age group of men between 19-34 year = 24.2 kg/m^2^, between 35-59 year = 26.3 kg/m^2^, between 60-74 year = 26.7 kg/m^2^ and above 75 year = 25.8 kg/m^2^. Due to the earlier retirement age of women, other groups were formed for them resulting these data: 19-34 year = 23.3 kg/m^2^, 35-54 year = 26.0 kg/m^2^, 55-74 year = 28.1 kg/m^2^ and above 75 year = 27.0 kg/m^2^. Within all age groups, the prevalence of overweight was 41.6% for men and 32.1% for women, while obesity was recorded in 11.6% and 18.1% of men and women, respectively.

A similar but smaller professional study was conducted between 1992 and 1994 measuring altogether *2,568* people of different ages. Among the *1,164* men examined, the prevalence of overweight was 41.9%, and that of obesity was 21.0%. Overweight was recorded in 27.9%, obesity in 21.1% of the *1,404* females who participated in the survey [4].

Ten years later, in a survey among incidentally involved primary care patients between 40-70 y of age, 45% prevalence of overweight and 32% of obesity was diagnosed, 3% of them with morbid obesity [6].

According to the WHO database, the prevalence of obesity was 26.2% among Hungarian males and 22.9% among females. Overweight was found 65.8% and 49.4% among males and females, respectively, increased by age, although the sources of data were not clearly described [1].

In 2009, a nationwide study targeted to select participants representatively, achieved only 35% response rate. Health workers measured the anthropometric parameters of *1,165* people. The means of BMI in the selected age groups of *men* were as follows: 18-34 y = 25.0 kg/m^2^, 35-64 y = 28.4 kg/m^2^ and over 65 y = 28.7 kg/m^2^. The same data for *women* were 23.6 kg/m^2^, 28.1 kg/m^2^ and 29.8 kg/m^2^, respectively. *Obesity* was diagnosed in 26.2% of men and in 30.4% of women, while morbid obesity was presented in 3.1% and 2.6%, respectively. *Abdominal* obesity was more prevalent among women than men (51.0% vs. 33.2%) and its ratio increased parallel with age in both genders [7, 8].

At the same time, another survey was conducted via the internet in various regions of Hungary involving *27,746* responders who filled the questionnaires. Among males, according to the self-measured data, *overweight* was reported by 31.5%, 43.5% and 49.6% in the age groups of 18-34 y, 35-64 y and over 65 y, respectively, while these figures were 17.8%, 33.6% and 39% among females. Within the same age categories, *obesity* was measured at 14%, 30.8% and 26.4% among men and at 12.5%, 27.4% and 28.4% among women. Irrespectively of age, self-measured *overweight* was reported by 40.6% of men and 30.5% of women, *obesity* by 25.0% and 23.6% [9].

A specific professional group was selected for a survey in 2002. Among *18,763* policemen and *2,037* policewomen (20-55 year), the prevalence of obesity was 19.1% and that of overweight was 43.8%. The distribution of waist circumference between 94-102 cm was 21.9%, above 102 cm was 22.6%. The ratio of obese officers increased with the age; it was 11.8% (between 20-25 year) and as high as 29.3% (between 40-45 year). Geographical differences were also recorded. Obesity was presented in 15.3% among policemen who served in the capital, and in 19.4% among those who were country-based [10].

### Aim

Since reliable, updated data of the Hungarian population are lacking, our main goal was to fit this gap. Collecting and presenting new data and comparing them to figures of the last wide range survey of 25 years before was aimed. Without governmental support, we approached primary care providers.

## Methods

Our study was conducted between September 2012 and April 2013, at primary care encounters and during community activities of primary health care staff including occupational health services as well, in all geographical regions in Hungary. Only non-institutionalized adults from 18 years of age were involved on a voluntary basis.

### Selection for subjects and criteria for exclusion

The participating family physicians (GPs) were given a detailed written description of methods for anthropometric measurements and asked to recruit the first 200 persons (over 18 y) visiting their office or persons who were seen by their community activities. They were asked to fill a written or electronic data sheet with the figures below.

Only those patients were excluded whose conditions might have been influenced by morbidities with weight consequences (cancer, COPD, cystic fibrosis, renal failure, pregnant and lactating women, etc.). Exclusion was decided by the GP.

### Body height [cm]

Persons on barefoot, measured with approved *stadiometer*, when head positioned in Frankfort plane (an imaginary line from lower border of the eye orbit to the auditory meatus).

### Body weight [kg]

Measured with a regularly calibrated weighing machine, in light clothing, fasting and with empty bladder, after defecation.

### Waist circumference [cm]

Using a professional tape (measured horizontally, halfway between the lower margin of ribs and iliac crest. Umbilical level was inacceptable).

All data were expressed in round figures.

The presence or absence of metabolic disease (*diabetes* and/or *hypertension*) was also registered.

### Educational level

The participants’ highest achieved level of education: not completed the 8-year elementary school (*under*), completed only elementary school (*primary*), graduated in secondary school and/or skilled worker qualification (*secondary*), having university or college degree (*higher*).

The WHO-established *BMI categories* were used in our survey (underweight <18.5 kg/m^2^, *normal* = 18.5-24.9 kg/m^2^, *overweight* = 25-29.9 kg/m^2^, *obese* >30 kg/m^2^).

Regarding *waist circumference*, the upper limit of *normal* range was 94 cm for men and 80 cm for women, the “*risky*” range was between 94-102 and 88-94 cm respectively, and *abdominal obesity* was diagnosed above these values.

#### Statistics

Descriptive statistics were done. Proportions were calculated with 95% confidence intervals. Univariate and multivariate logistic regression models were created to check the associations between the outcome and its influencing factors. Odds ratios were reported with their 95% confidence intervals. Statistical analyses were performed in *Stata 9.2*.programme.

## Results

Altogether, the data of *41,163 subjects* were collected and *40,331* (16,544 men and 23,787 women) were analyzed, because of missing or incomplete records.

Coming from all parts of Hungary, the number of participating GPs was 244, representing 3.5% of all primary care practices. The location and number of practices were as follows: Budapest, the capital of Hungary: 38, cities and towns: 99, and villages: 107.

The Tables 1 and 2 present the most important data of prevalence and distribution of BMI and waist circumference in our recent study, compared with the same age categories of the survey in 1985-1988 [3]. Visible shift toward higher BMI groups can be observed in all categories.

**Table 1:** **BMI**

	Men [%]					Women [%]			
	Age [y]	Underweight	Normal	Overweight	Obese	Underweight	Normal	Overweight	Obese
Recent	18-29	4.9	54.1	27.8	13.2	11.8	61.2	15.9	11.1
	30-39	0.8	33.2	41.7	24.3	5.1	50.9	23.9	20.1
	40-49	1.1	24.4	41.2	33.3	2.2	40.7	28.4	28.7
	50-59	0.6	21.3	39.5	38.6	1.1	27.3	35.3	36.6
	60-69	0.6	16.4	43.0	40.1	0.8	21.2	36.4	41.6
	70<	0.6	21.4	46.1	31.9	0.9	23.7	38.2	37.2
	18-34	3.6	47.7	32.5	16.2	10.0	58.4	17.8	13.8
	35-54					2.3	39.1	29.7	29.0
	35-59	0.7	24.0	40.6	34.7	1.9	35.4	31.4	31.3
	55<					0.9	23.2	36.9	39.0
	60<	0.6	18.5	44.3	36.7				
*Former*	*18-34*	*5.7*	*57.1*	*32.2*	*4.9*	*18.4*	*55.5*	*19.8*	*6.3*
	*35-54*					*5.1*	*42.0*	*35.0*	*17.6*
	*35-59*	*3.1*	*34.0*	*48.2*	*14.2*				
	*55-74*					*2.1*	*23.1*	*41.8*	*32.1*
	*60-74*	*3.4*	*29.8*	*49.3*	*17.5*				
	*75<*	*5.9*	*35.9*	*45.9*	*12.4*	*4.0*	*33.0*	*40.0*	*22.9*

Distribution of individuals in the different BMI categories regarding age periods and decades (in percent). Comparison between recent and *former* data [3].

**Table 2:** **Means of BMI**

		Men	Women
	Age [year]	Mean (±SD) [kg/m ^2^]	Mean (±SD) [kg/m ^2^]
Recent	18-29	24.7 (±4.8)	23.23 (±5.2)
	30-39	27.3 (±4.8)	25.49 (±5.8)
	40-49	28.5 (±5.4)	27.17 (±5.9)
	50-59	29.1 (±5.3)	28.65 (±5.7)
	60-69	29.3 (±4.9)	29.34 (±5.4)
	70<	28.4 (±4.5)	28.64 (±5.2)
	18-34	25.5 (±4.9)	23.86 (±5.5)
	35-59	28.6 (±5.3)	
	35-54		27.24 (±5.9)
	55<		28.97 (±5.4)
	60<	28.9 (±4.7)	
*Former*	*18-34*	*24.2*	*23.3*
	*35-54*		*26.0*
	*35-59*	*26.3*	
	*55-74*		*28.1*
	*60-74*	*26.7*	
	*75<*	*25.8*	*27.0*

Distribution in different age periods. Comparison between recent and *former* data [3].

Increase in waist circumference, already in the younger generation is visible among figures of the Table 3.

**Table 3:** **Waist circumference**

	Men			Women		
Age [y]	Normal ***<94 cm***	Risky ***94-102 cm***	Abdominal obesity ***102 cm<***	Normal ***<80 cm***	Risky ***80-88 cm***	Abdominal obesity ***88 cm<***
18-29	72.6	14.1	13.3	54.6	19.5	25.9
30-39	50.5	23.2	26.3	37.3	21.0	41.7
40-49	38.9	25.1	36.0	25.9	19.9	54.2
50-59	31.3	25.6	43.2	14.8	16.4	68.8
60-69	26.0	25.5	48.5	9.3	13.9	76.8
70 <	29.2	28.2	42.6	12.1	13.0	75.0

Distribution of men and women in established categories of abdominal obesity (normal, cardiovascular risk and abdominal obesity) in percent.

According to waist circumference, parameters of risk were identified among more men in rural than urban settings. In their fourth decade of life (30-39 year), the ratio of males at risk was much higher (20.0% vs. 26.8%) in the rural population and in the fifth decade (40-49 y) it was still higher (31.5% vs. 36.4%). A similar trend was observed among rural women (38.2% vs. 54.9%) in their forties and (63.8% vs.75.5%) above 70 (years of age).

Table 4 shows the distribution of BMI and waist circumference categories regarding different levels of education. Fewer people with higher education were represented in the *obese* category. Men with a *higher* degree had the highest proportion among the *overweight* BMI group and also higher in the normal then in obese group, while females had the lowest record among the *obese* and the highest in the *normal* range. Similar results were found according to waist circumference. In the categories of obesity (regarding BMI and waist circumference) there were inverse relations between odds ratios and degrees of education.

**Table 4:** **Educational level, BMI and waist circumference**

	Men [%]						
Category	BMI [kg/m ^2^]				Waist circumference [cm]		
	Underweight	Normal	Overweight	Obese	Normal	Risky	Abdominal obesity
**Education**							
Under	2.0	30.1	39.7	28.3	39.5	23.2	37.3
OR (95% CI)	1.04 [0.54-2.02]	1.12 [0.91-1.37]	1.19 [0.99-1.44]	0.74* [0.61-0.91]	1.06 [0.88-1.28]	0.99 [0.80-1.24]	0.94 [0.78-1.14]
Primary	1.9	27.8	35.7	34.7	38.1	23.2	38.8
OR (95% CI)	1.00	1.00	1.00	1.00	1.00	1.00	1.00
Secondary	1.1	24.9	40.7	33.3	37.9	23.8	38.4
OR (95% CI)	0.58* [0.42-0.79]	0.86* [0.79-0.94]	1.24* [1.15-1.34]	0.94* [0.87-1.02]	0.99* [0.92-1.07]	1.03* [0.94-1.13]	0.99* [0.91-1.07]
High	0.6	28.0	45.3	26.1	40.6	25.7	33.8
OR (95% CI)	**0.31** [0.17-0.55]	1.01* [0.90-1.14]	1.50* [1.35-1.67]	0.66* [0.59-0.74]	1.11* [0.99-1.23]	1.15* [1.02-1.29]	0.81* [0.72-0.89]
	**Women** [%]						
Under	3.1	26.1	29.8	41.0	17.0	12.4	70.6
OR (95% CI)	1.15* [0.81-1.65]	1.01* [0.88-1.16]	0.87* [0.76-0.99]	1.11* [0.98-1.26]	1.09* [0.93-1.29]	0.87* [0.73-1.05]	1.02* [0.89-1.16]
Primary	2.7	25.9	32.8	38.5	15.7	14.0	70.3
OR (95% CI)	1.00	1.00	1.00	1.00	1.00	1.00	1.00
Secondary	3.1	37.1	31.6	28.1	24.3	18.4	57.3
OR (95% CI)	1.16 [0.96-1.40]	1.68* [1.57-1.80]	0.95 [0.88-1.01]	0.63* [0.59-0.67]	1.73* [1.59-1.88]	1.38* [1.27-1.52]	0.57* [0.53-0.61]
High	2.7	50.2	26.8	20.3	32.3	20.3	47.5
OR (95% CI)	1.01 [0.78-1.31]	2.88* [2.63-3.15]	0.75* [0.68-0.82]	0.41* [0.37-0.45]	2.55* [2.31-2.82]	1.56* [1.39-1.75]	0.38* [0.35-0.42]

Distribution of participants according to different levels of education, different groups of BMI, and waist circumference [in percent]. Odds ratios (OR) and confidence intervals [95% CI] with reference to *primary* level of education. Significance is marked with bold and *.

In both genders, these differences were mostly statistically significant when comparing primary and other educational levels.

The prevalence data for *obesity* was the highest in the villages (35.4%), the lowest in Budapest (28.9%). *Overweight* was “only” (33.9%) in the villages, the highest in Budapest (37.1%) and 35.8% in the cities. Persons within *normal* BMI range were only 31.8% in Budapest. They represented the 32.5% of other urban and 28.6% of rural population.

As presented in Table 5 the incidence of registered metabolic morbidities (hypertension and diabetes) was different regarding type of domiciles, showing an inverse correlation with the number of inhabitants. The lowest was in Budapest and the highest in rural settings. These morbidities were also significantly correlated with levels of education and categories of BMI, with strong relation to the increasing age.

**Table 5:** **Morbidities**

Category	Women					Men				
	OR	Std. error	P > z	95% CI		OR	Std. error	P > z	95% CI	
**Ref.: Budapest**	*1.00*					*1.00*				
Cities	**1.278**	**0.093**	**0.001**	**1.107**	**1.473**	1.107	0.088	0.201	0.947	1.295
Villages	1.071	0.080	0.356	0.925	1.239	1.052	0.086	0.532	0.895	1.237
**Ref.: primary**	*1.00*					*1.00*				
Under	1.018	0.074	0.810	0.881	1.174	1.075	0.110	0.482	0.878	1.315
Secondary	**0.471**	**0.017**	**<0.001**	**0.438**	**0.505**	**0.629**	**0.027**	**<0.001**	**0.578**	**0.685**
High	**0.308**	**0.015**	**<0.001**	**0.279**	**0.340**	**0.620**	**0.035**	**<0.001**	**0.554**	**0.694**
**Ref.: normal**	*1.00*					*1.00*				
Underweight	**5.354**	**0.216**	**<0.001**	**4.946**	**5.795**	**5.354**	**0.264**	**<0.001**	**4.859**	**5.899**
Overweight	**2.685**	**0.100**	**<0.001**	**2.496**	**2.888**	**2.470**	**0.110**	**<0.001**	**2.263**	**2.696**
Obese	**0.372**	**0.041**	**<0.001**	**0.298**	**0.461**	**0.623**	**0.107**	**0.006**	**0.443**	**0.874**

Odds ratios (OR), level of significance and 95% confidence intervals (CI) for metabolic diseases (hypertension or/and diabetes) by different type of domiciles, by different levels of education and BMI categories. Significance is marked with bold.

## Discussion

### Main findings

Comparing the data of a quarter century ago, the BMI has become higher in all age categories and the distribution of the population also tended toward being overweight, moreover obese, resulting a 2-4-fold increase in the percentage of incidence in some age groups. This shift has been more prominent among males. Their BMIs have been higher from the middle decades, while earlier women used to have larger surplus, which means rearrangement between categories, a shift from being overweight to becoming obese.

Significant differences were found between the educational level and BMI categories. Although there were fewer obese persons among the subjects with higher education, being overweight was common, while women with a higher degree were less obese.

Registered metabolic morbidities were strongly correlated with BMI and both were inversely related to the level of urbanization.

### Comparison with previous research

Previous Hungarian studies used different methods and other age categories for the selection of participants; therefore it was often difficult to make proper comparison. Most of these studies had a much lower number of participants [4, 6–10]. At the time of the first nationwide survey 25 years ago, Hungary still had 10.6 million inhabitants. Individuals included in the study represented *0.16%* of the whole population [3]. Currently, with the number of the population decreasing, our study has achieved a *0.53%* participation rate in the age-cohort over 18 years. This ratio is much higher than that of the similar surveys in other countries. Data regarding education and waist circumference have not been compared or analyzed before. Another problem emerged, because the distribution of data within different age-cohort regarding gender was not identical. Earlier, the official retirement age was 55 years for women and 60 years for men. We tried to present our data considering the grouping of this survey as well. Previously, 20 kg/m^2^ was used as a lower threshold of *normal* BMI-group, but the WHO has recently introduced the limit of 18.5 kg/m^2^.

Comparing our data to that of neighboring countries, these figures are higher although they were given almost within the same range in the WHO database [1, 11]. In Austria, in the Western neighbor country, in the self-reported data from 5 surveys was compared, analyzing the decade-long trend in the incidence of obesity. In 2007, the prevalence of overweight was higher among men than women (46.3% vs. 31.2%). There was a clear east–west gradient for obesity in both sexes; the highest figures were found in Eastern Austria: 18.1% for women and 16.1% for men [2]. There was also a higher prevalence of obesity in the former Soviet-bloc (socialist) countries. It could be explained by many similar social and economic reasons as well [1, 2].

Although Hungary is situated mainly in lower terrains (the highest mountain is 1014 m above sea level) there were only small differences (100-300 m) regarding altitudes between geographic locations of GPs. It is not surprising that data were mainly similar without such differences as presented in a study from the US, with lower registered BMI of persons living in the mountains [12]. Findings were similar regarding to the higher incidence of metabolic morbidities among rural population. Urbanization and obesity prevalence exhibited an inverse relationship.

There was no more comparison to available data of other countries worldwide, while almost all of them have to face to the “*obesity pandemic*” [1, 13].

People living in smaller settings may have many disadvantages regarding access to healthcare delivery. This reason combined with their lifestyle, which is often not appropriate could be the reason for higher incidence rate of metabolic morbidities and overweight/obesity.

Requirement regarding representativeness of educational level was properly fulfilled. Comparing the population at large in Hungary and that of the study, *under-*educated were both 5%, *primary* and *secondary* schools were completed by (27% vs. 33%) and (51% vs. 46%) and (14% vs. 15%) had *higher* educational degree [5].

### Limitations and strengths

The participants of this nationwide survey were mainly primary care patients and less from community settings. Without appropriate registration, the real ratio is unknown. It might be the reason why the peak of the age tree of participants was almost 10 years higher than that of the population at large. Patients with cardiovascular morbidities might have been overrepresented, although prevalence of the 2 registered metabolic diseases (hypertension and diabetes) in the older generation of men was closer to the data of national morbidity register (below 35 y: 13.5% vs 18%, between 35-65 y: 71% vs 76%; above 65 y: 79% vs 82%) [5, 14]. There were similar data by female as well. The very simply process of randomization (the first persons), the high number of participants and data presentations according to smaller age groups (life-decades), and to waist circumference, which were not measured previously, could counterbalance this possible bias.

Although anthropometric data were measured by the medical staff, individual inaccuracy could also be encountered, but it could be balanced during under- and over recording.

## Conclusions

Primary care settings can be a proper place to register and follow up anthropometric parameters and family physicians should be motivated to perform it continuously. Early medical intervention or even advices can help in the prevention of obesity. Proper under- and postgraduate education and use of updated guidelines could be a professional help in daily practices of family physicians, not only in Hungary [15, 16].

Beside the involvement of health workers, much more governmental support, population awareness are needed and clear health policy recommendations should be outlined.
